# The diagnosis and management of small and indeterminate lymph nodes in papillary thyroid cancer: preoperatively and intraoperatively

**DOI:** 10.3389/fendo.2024.1484838

**Published:** 2024-11-14

**Authors:** Ang Hu, Jiahe Tian, Xinpei Deng, Zhongyu Wang, Yin Li, Jianwei Wang, Longzhong Liu, Qiuli Li

**Affiliations:** ^1^ Department of Head and Neck Surgery, State Key Laboratory of Oncology in South China, Guangdong Provincial Clinical Research Center for Cancer, Sun Yat-sen University Cancer Center, Guangzhou, China; ^2^ Department of Urology, State Key Laboratory of Oncology in South China, Guangdong Provincial Clinical Research Center for Cancer, Sun Yat-sen University Cancer Center, Guangzhou, China; ^3^ Department of Ultrasound, State Key Laboratory of Oncology in South China, Guangdong Provincial Clinical Research Center for Cancer, Sun Yat-sen University Cancer Center, Guangzhou, China

**Keywords:** thyroid cancer, diagnosis, lymph node metastasis, small and indeterminate lymph nodes, management

## Abstract

Although thyroid cancer is an indolent tumor with a favorable prognosis, lymph node metastasis (LNM) serves as a major concern for many patients. Because LNM is strongly correlated with recurrence, distant metastasis, and shortened survival, a precise and timely diagnosis and following appropriate management for LNM are necessary. However, significant challenges still exist in the diagnosis of small LNs (<1 cm in diameter), and their low volume makes it difficult to determine whether they are metastatic or benign. Therefore, the diagnostic technique for these small and indeterminate LNs (siLNs) has been one of the leading research subjects in recent years. The implementation of innovative technologies, such as contrast-enhanced ultrasonography, frozen section, and molecular detection, has brought great progress to the diagnosis of siLNs. Meanwhile, the strategies for managing siLNs in clinical practice have evolved considerably over the past several years, with several appropriate options recommended by guidelines. In this review, we aim to provide a systematic overview of the latest studies and potential evidence about effective approaches for detecting and evaluating siLNs. Furthermore, the following management modalities of siLNs in different situations are well discussed.

## 1 Introduction

The incidence of thyroid cancer (TC), the most common endocrine malignancy, has increased in recent years, mostly attributed to more common imaging techniques and more universalized surveillance practices. In 2020, its incidence rate was 4.9%, ranking 9th for malignant tumors of both sexes, while its mortality rate was much lower, with rates of 0.5 per 100,000 in women and 0.3 per 100,000 in men (1). TC encompasses several pathological subtypes ranging from the most indolent to the most malignant in terms of clinical behavior, with papillary thyroid cancer (PTC) accounting for approximately 90% of all cases (2). The 5-year survival rate of patients with TC is approximately 84.3% in China, whereas it reaches as high as 98% in Korea and the United States. This disparity can be attributed to the greater nationwide average popularity of standardized treatment (including timely and proper lymphatic management) for PTC in developed countries (3–5). Approximately 20–90% of adult PTC patients exhibit lymph node metastasis (LNM), which is closely associated with increased rates of recurrence and reduced survival (6–8). Therefore, performing lymph node dissection (LND) for confirmed or suspicious LNM is frequently necessary, suggesting that the diagnosis and management of lymph nodes (LNs) are highly important in the treatment of PTC patients.

Modern medical images have demonstrated good performance in detecting cervical LNs in PTC patients, in which both ultrasound (US) and computer tomography (CT) have achieved high levels of sensitivity and specificity, surpassing 80% (9). However, encouraging results are derived from statistics that include nodes of all diameters. Small and indeterminate lymph nodes (siLNs) are described as LNs with a diameter less than 1.0 cm (8mm in certain publications), and ascertaining their nature is often arduous. In fact, diagnostic challenges are primarily present in siLNs. During the physical examination, almost all of siLNs were unpalpable nodules. The metastatic imaging features of small LNs are usually not typical and are less common on conventional US and CT. The low-volume characteristic of small and indeterminate LNs (siLNs) also adds to the difficulty in performing gold standard diagnostic technique, fine needle aspiration (FNA). In addition, multiple siLNs are usually observed at the same time, so it is not feasible to puncture each one. Clinically, accurate determination of nodal status is a crucial part of appropriate surgical treatment planning. Due to the uncertainty of siLNs, selecting the right management measure following preoperative examinations remains controversial, without solid guidelines about optimal management. Therefore, the existence of siLNs is an issue worth exploring in depth, and numerous studies have been undertaken to address this matter. Based on published reports and potential current evidence, for the first time, we have presented an up-to-date summary of the viable and underlying methods for diagnosing siLNs and then discussed various strategies for managing siLNs, from aggressive treatment to a more conservative approach.

## 2 Preoperative diagnosis

### 2.1 Conventional US and contrast-enhanced US

**Figure 1:**
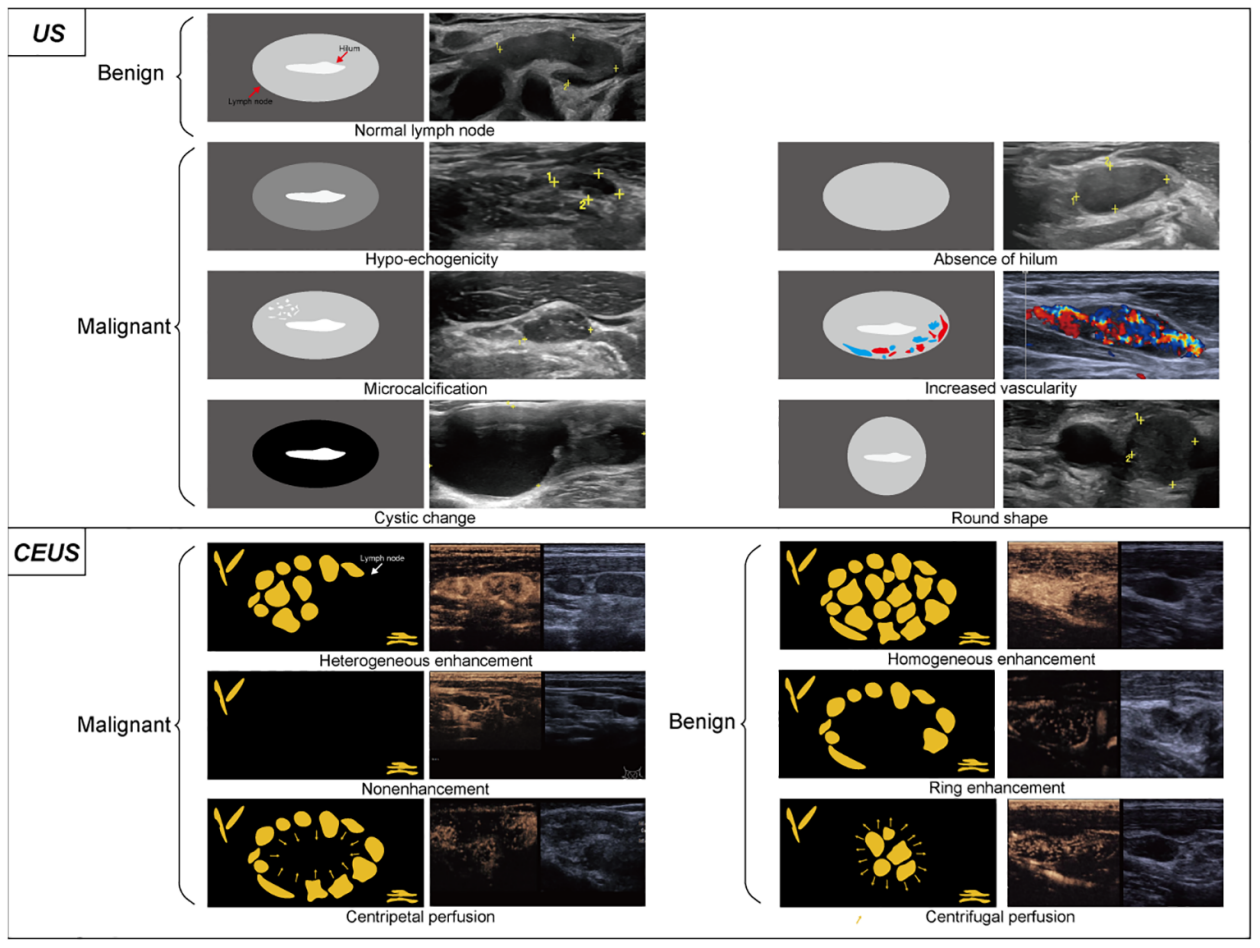
Conventional ultrasound and contrast-enhanced ultrasound image characteristics of LNs. On conventional ultrasound, ultrasonic malignant manifestations generally includes hypo-echogenicity, absence of hilum, microcalcification, increased vascularity, cystic change and round shape. Thyroid imaging reporting and data system (TIRADS) scores each thyroid nodule and differentiate benign and malignant based on the presence of these signs. Among them, the calcification exhibits a relatively higher degree of malignant potential. In contrast-enhanced ultrasound, heterogeneous enhancement, nonenhancement, and centripetal perfusion are the main manifestations of malignant LNs, while homogeneous enhancement, ring enhancement, and centrifugal perfusion are the main manifestations of benign LNs.

US continues to be the predominant imaging modality for the assessment of metastatic cervical adenopathy in PTC patients and serves as the major tool for both preoperative evaluation and follow-up examination (10). Currently, several US features of LNs are predictive of malignancy, including hypo-echogenicity, absence of hilum, cystic changes, round shape, increased vascularity, and microcalcifications (**Figure 1**) (10, 11). However, the use of US alone is insufficient for the assessment of cervical LNM (12, 13). Previous reports have indicated that the efficacy of conventional US is disputed due to its inconsistent sensitivity, which ranges from 0.33 to 0.7 (14). Contrast-enhanced US (CEUS) is a highly effective method for examining tissue vascularity compared to conventional ultrasound (15). The application of this technique has demonstrated great efficacy in accurately distinguishing between malignant and benign LNs in PTC (16–18). Among the preoperative sonographic features of CEUS, heterogeneous enhancement, nonenhancement, and centripetal perfusion are independent ultrasonographic characteristics of malignant LNs, while homogeneous enhancement, ring enhancement, and centrifugal perfusion are those of benign LNs (**Figure 1**) (19, 20). In 2023, two publications showed that significant progress was made in the field of CEUS-based LN imaging, especially in facilitating the precise identification of siLNs (20, 21). In the first article, Zhang et al. (20) included 64 participants with suspected PTC (76 LNs in total) and compared the diagnostic performance of CEUS and US for lymphatic metastasis. The sensitivity, specificity, and accuracy of the CEUS group were 16%, 10%, and 3% greater than those of the US group, respectively. Through further careful exploration of the association between diagnostic performance and LN size and location, they discovered that CEUS exhibited superior diagnostic accuracy when the LNs were measured 1 cm or smaller and were located in the central neck region. The second study specifically investigated the value of CEUS in the identification of siLNs. Of note, siLNs were defined as those with short-axis diameters less than or equal to 8 mm rather than 1 cm. This research performed a comprehensive evaluation of these LNs in the vascular and postvascular phases separately. This evaluation indicated that the presence of perfusion defects during the vascular phase exhibited a significantly high level of specificity, reaching 96%. Additionally, the absence of iso-enhancement in the postvascular phase demonstrated a high level of accuracy, particularly 93%, as well as a negative predictive value of 100%. Finally, it was confirmed that the combined model of US and postvascular phase features yielded better results than did the model that relies solely on US features for the purpose of diagnosing potentially malignant small lateral cervical LNs (AUC 0.94 vs. 0.73; P <.001) (21). Two reports utilized the same contrast agent, Sonazoid, owing to its advantageous characteristic of providing a distinct postvascular phase (Kupffer phase) (22). In contrast, a distinctive difference of them was the method used to inject Sonazoid; namely, the former directly injected the agent into the middle lobe of the thyroid, while the latter was an intravenous bolus injection. The benefit of intrathyroidal injection lies in the agent being absorbed by sentinel LNs, similar to sentinel LN mapping, resulting in more precise and targeted detection of metastatic LNs. Moreover, both articles, interestingly, were published on the same day.

Even so, a subsequent investigation revealed the suboptimal performance of CEUS in terms of diagnostic sensitivity for LNM within the diameter ranges of 0–5 mm and 5–10 mm, with sensitivities reaching only 61.0% and 66.6%, respectively (23). One potential explanation could be attributed to the use of another contrast agent, SonoVue, in this study. A detailed comparison of the difference between SonoVue and Sonazoid can be found in another review (24). At times, the effectiveness of CEUS is limited due to certain inherent sonographical defects. One difficulty is differentiating between the subtle enhancements in tiny metastatic foci and the surrounding lymphatic parenchyma, which is primarily associated with the indolent characteristic of PTC (17). Another explanation is that homogenous and iso-enhancement can be observed in both benign and metastatic lymph nodes (25).

### 2.2 Computed tomography

Following US, CT is recommended by the ATA2015 guideline to serve as a secondary option for imaging the status of cervical LNs (9, 10). When utilizing conventional CT, it is also challenging to diagnose LNs that measure less than 1 cm. Therefore, Liu et al. (26) developed a parameter model based on dual-source dual-energy CT at a small field of view for the detection of small LNMs, which showed superior diagnostic accuracy compared to that of the CT image model. When combined with US, Zheng et al. (27) also constructed a CT-based deep learning model for the diagnostic assessment of suspicious lateral LNs. As reported, its diagnostic performance is comparable to that of FNA and experienced radiologists. In addition, dual-energy CT parameters could complement US in identifying siLNs (28). The main obstacles associated with CT images mostly stem from the limited diagnostic value of known metastatic signs. In this regard, a recent report has assessed the diagnostic efficacy of several contrast-enhanced CT features from the K-TIRADS guidelines within siLNs. The results demonstrated that the removal of “heterogeneous enhancement” from the K-TIRADS LN classification led to a higher specificity and positive predictive value than before (29). Additionally, Lee et al. (30) investigated the predictive accuracy of CT for detecting cervical lymphatic micrometastasis in PTMC patients who had opted for active surveillance (AS). They found that CT displayed a better diagnostic performance in detecting cervical LNM than US and that CT provided additional benefit after the evaluation of cervical LNs by US. Regardless of the individual effectiveness of CT or US, most reports on the use of dual imaging, including both CT and US, have shown that the detection rate has increased markedly (31). Nonetheless, in this study, the combination of US and CT did not improve diagnostic accuracy compared with the use of CT alone, which is distinct from what was stated previously. The likely reasons for this difference are the individuality of the LN status and the unique patient selection criteria for AS. Hence, it should be noted that the conclusions were applicable only to this specific scenario. In summary, further studies are warranted to identify additional dimensions in which CT has superior efficacy; otherwise, CT will be used as an auxiliary approach for US most of the time.

### 2.3 Fine-needle aspiration

Guidelines strongly recommend FNA for the diagnosis of siLNs, of which fine needle aspiration cytology (FNAC) and fine needle aspiration thyroglobulin (FNA-Tg) are two popular techniques (10, 32). A recent meta-analysis, which included twenty-one studies and pooled 1662 malignant and 1279 benign LNs from 2712 patients, revealed that FNAC had a greater level of specificity than FNA-Tg, while FNA-Tg showed greater sensitivity than FNAC (33). Moreover, the study concluded that the combination of FNAC and FNA-Tg, whether used preoperatively or postoperatively, could achieve better diagnostic performance than either method alone. In mainstream guidelines from seven international societies, including the American College of Radiology (ACR), ATA, American Association of Clinical Endocrinologists (AACE), ETA, KTA, National Comprehensive Cancer Network (NCCN), and SRU, only those of the ATA, KTA, SRU, and ETA provided the detailed US characteristics and LN size that can be used to determine the optimal timing for conducting FNA of suspicious LNs (**Table 1**) (10, 32, 34–38). According to KTA, the minimum achievable size for FNA is 3 mm. However, some experts believe that FNAC is indicated only for LNs larger than 5 mm to obtain an adequate number of cells for a more reliable diagnosis, which is consistent with the recommendations of other guidelines except for KTA (39). Clinically, when the diameter of the LN is less than 10 mm, it is challenging to puncture the center of the lesion; thus, the procedure is best completed by skilled radiologists and cytologists to increase the success rate (40, 41). Hydrodissection is a promising technique used to separate target lesions from nearby essential structures for the ablation of cervical lesions. At present, hydrodissection is being applied in FNA procedures. Cheng et al. (42) evaluated the safety and effectiveness of hydrodissection-assisted small LN biopsy by calculating the separation success rate of hydrodissection, the technical success rate, and the histopathologic success rate. According to their results, all of these related indicators achieved an astonishing 100%. Therefore, they established that hydrodissection enabled the safe and efficient performance of US-guided biopsy of subcentimeter cervical LNs near important arteries. Chen et al. (41) reviewed the medical records of 996 suspicious lateral LNs aspirated 2 to 3 times and classified them into two groups based on their respective sizes (large group and small group). They observed that the incidence of malignancy in larger LNs was marginally greater than that in smaller LNs, whereas LN size was not an independent predictor of malignancy according to multiple regression analysis. In contrast, there are several paradoxical results regarding the predictive role of LN size, whereas other studies have identified it as a predictor (36, 43, 44). Therefore, we should understand that being tiny may not necessarily indicate a reduced likelihood of malignancy. Notably, patients undergoing FNAC have a 0.5% cumulative incidence of needle tract implantation (45). It suggested that mindlessly increasing aspiration times or expanding puncture indications was not advised, despite this issue often being overlooked in previous clinical practice.

**Table 1:** Indications for FNA biopsy recommended by classic guidelines for LNs (10, 32, 35, 36).

Guideline	Indication for US performance	Indication for size cutoff
ATA2015	Microcalcifications, cystic aspect, peripheral vascularity, hyper-echogenicity, round shape	≥8–10 mm in the smallest diameter
	Microcalcifications, cystic aspect, peripheral vascularity, hyper-echogenicity, round shape + node growth/adjacent to vital structures	<8–10 mm in the smallest diameter
KTA	Cystic change, calcification(micro/macro), hyper-echogenicity (focal or diffuse), abnormal vascularity (peripheral or diffuse)	Short diameter of suspicious lymph node > 3–5 mm
	Loss of central hilar echo and absence of central hilar vascularity	Short diameter of indeterminate lymph node > 5 mm
ETA	Microcalcifications, partially cystic appearance, peripheral or diffusely increased vascularization, hyper-echoic tissue looking like thyroid	> 10 mm or documented growth
SRU	Heterogeneous echotexture, calcifications, cystic areas, round shape, mass effect	≥7 mm in the short axis

ATA2015, 2015 American Thyroid Association Management Guidelines; ETA, European Thyroid Association; KTA, Korean Thyroid Association; SRU, Society of Radiology in Ultrasound.

Ever since Pacini et al. first presented a method in which FNA was combined with thyroglobulin washout, this method has proven to have outstanding specificity and selectivity in clinical practice (46–48). Currently, FNA-Tg has the ability to detect and identify indeterminate LNs with a diameter as small as 5 mm (49). However, the primary concern in the current FNA-Tg technique revolves around the determination of a universally applicable and optimal cutoff value (47). Recent studies have revealed a collection of Tg thresholds and their corresponding indicators, encompassing sensitivity, specificity, and accuracy. For instance, Zhu et al. (50) analyzed a total of 22 studies and performed a subgroup analysis of patients according to the cutoff value; the results confirmed that the threshold of 1 ng/mL had the maximum sensitivity, while 40 ng/mL had the highest specificity. The measurement of TgAb via FNA is also a feasible way to detect the presence of metastatic LNs (i.e., FNA-TgAb). Nonetheless, compared with FNA-Tg, FNA-TgAb exhibited poorer performance in identifying LNs with a diameter less than 10 mm (51). Indeed, it is well recommended to utilize FNA-Tg at any FNA facility, particularly when LNs are low in volume (50). Furthermore, the latest studies have suggested several points that need to be considered during clinical application: (1) FNA-Tg should be interpreted with caution only if the serum Tg concentration exceeds 10 ng/ml (47); (2) the absolute Tg content may be a potential alternative that could provide a more objective expression of Tg washout (52); and (3) FNA-TG washout may be particularly valuable for diagnosing cystic or highly vascular LNs, as these types of LNs may yield negative FNAC results (53). In addition to medical imaging, FNA serves as the most commonly used approach because of its considerable sensitivity and specificity. Along with its associated techniques, such as FNAC and FNA-Tg, FNA has significant potential for making further advancements in diagnosing siLNs (54).

## 3 Intraoperative diagnosis

### 3.1 Sentinel lymph node biopsy

**Figure 2:**
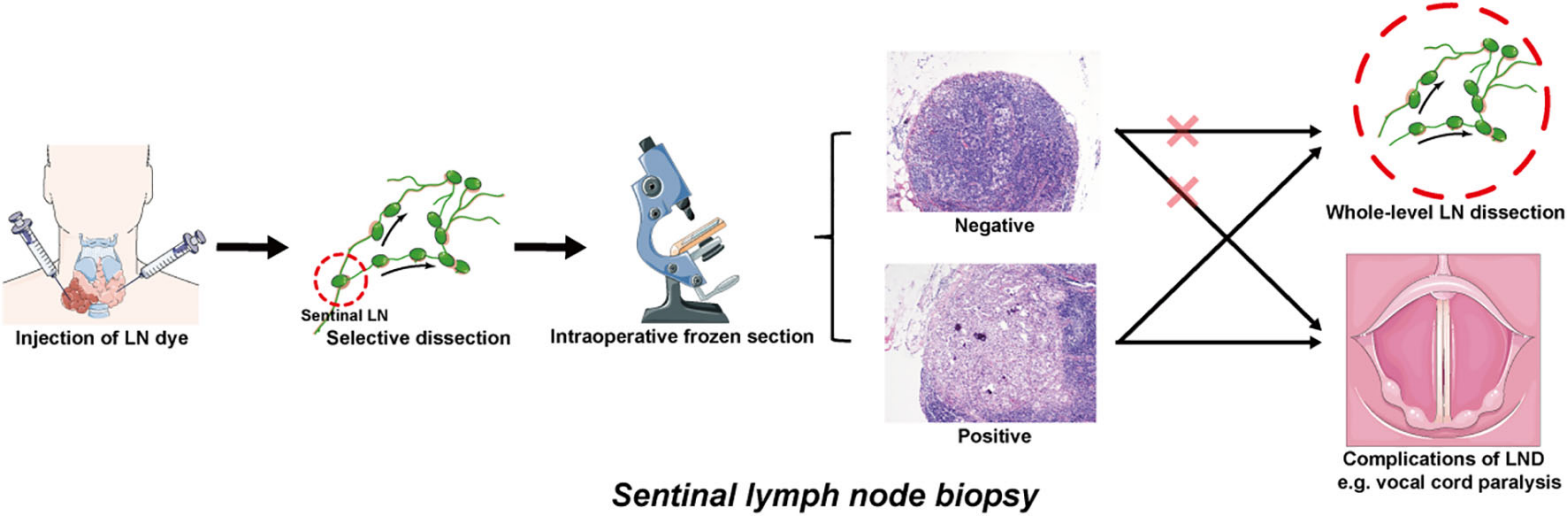
Operational pattern of SLNB for detecting the siLNs of PTC patients. The sentinel lymph nodes are identified by injecting appropriate LN dyes. The colored LNs are extracted from the suspect compartment and sent to frozen section, while the uncolored LNs are temporarily retained. If the frozen pathology results are negative, it is feasible to dispense with further dissection and preserve the remaining portion of the LNs. If positive, whole-level LND is indicated, which may be accompanied by potential surgical complications (e.g., vocal cord paralysis induced by recurrent laryngeal nerve injury). siLNs, small and indeterminate lymph nodes; SLNB, sentinel lymph node biopsy; PTC, papillary thyroid cancer; LN, lymph node; LND, lymph node dissection.

Facing the difficulties in identifying and dissecting small LNs with indeterminate imaging indications of metastasis, sentinel lymph node biopsy (SLNB) has attracted increasing amounts of attention from oncologists and clinicians because of its high detection rate and minimal invasiveness. In addition to being initially used in melanoma and breast cancer, SLNB was also applied to detect indeterminate metastatic LNs in the TC and acts as an alternative to replace cervical LND (55). As the main tumor drainage LNs to which LNM first occurs, sentinel lymph nodes were marked with the help of specific LN dyes. The stained LNs were removed from the suspicious compartment, while the unstained LNs were reserved. Selective removal of stained sentinel LNs effectively eliminates the uncertainty of random sampling, and the subsequent frozen pathology reliably verifies whether LNM occurs at this level. In this way, SLNB provides an accurate reflection of the status of the remaining LNs, particularly when some of them appear as siLNs (56). In the event that the SLNB yields a negative result, siLNs within the remaining portion of LNs can be confirmed to be benign, regardless of whether some siLNs are included in the sampled portion. Afterward, radical whole-level LND of the suspicious compartment and underlying surgical complications were avoided; otherwise, the opposite was true (**Figure 2**). **Table 2** presents a comparative analysis of SLNB studies that employed different modalities over the past five years. It was reported that an astonishing 100% detection rate of SLNB could be achieved, while the false-negative rate was comparable to approximately 20%, or even as high as 40% (55, 57). Combined with imaging examination, Liu et al. (58) recently proposed a new sentinel LN technique that uses conventional US and CEUS to assign every targeted sentinel LN a score (range from 0–5). The results demonstrated that the sensitivity and specificity could reach 97.1% and 93.9%, respectively, at a cutoff of 4 or higher.

**Table 2:** Comparison of SLNB studies over the past 5 years.

Method	Kind of study	Num	Spec	FNR (%)	SDR	Acc	Year
LS	Prospective study	96	80%	20%	67%	98%	2020 (57)
LS	Prospective study	55	100%	13.79%	96.36%	92.45%	2022 (60)
LS	Prospective study	24	NA	11.1%	95.8%	NA	2020 (59)
LS	Prospective study	38	100%	3%	94%	97%	2018 (56)
Carbon nanoparticle	Prospective study	78	98.7%	12.9%	98.7%	93.6%	2022 (62)
VD/LS/LS +VD/LS+SPECT	Meta–analysis	45	NA	0–38% /0–40% /0–17% /7–8%	83%/96%/87%/93	NA	2019 (115)
Methylene blue dye	Retrospective study	153	96.7%	14.3%	91.8%	94.3%	2020 (116)
Superparamagnetic iron oxide nanoparticles	Retrospective study	20	NA	0%	80%	NA	2019 (96)

VD, vital dye; LS, 99mTc–nanocolloid planar lymphoscintigraphy plus hand–held gamma probes.

Several related reports have been published presenting controversial results regarding the benefit of SLNB. On the one hand, SLNB was shown to have a low identification rate and only moderate sensitivity in the study by Albers et al. (57), who suggested that the concept of the sentinel LN as the first LN is not fully applicable to TC due to the interconnected communication of lymphatic vessels within and outside the thyroid gland. Therefore, the reports conclude that SLNB is inappropriate as an adjunct to surgery within the framework of contemporary PTC treatment concepts. On the other hand, some studies employing the same methods as those of Albers et al. have suggested the opposite findings, where SLNB was still considered to be a reliable method with excellent diagnostic accuracy, as least in the early stage of PTC (cT1–2, cN0) (59, 60). Past studies have focused mainly on the utility of SLNB in central compartment LNs, whereas SLNB has also begun to emerge as an effective diagnostic procedure for lateral compartment LNs in papillary and medullary TC (61, 62). Furthermore, one publication noted that the administration of activated charcoal suspension could aid in the visual confirmation of small and non–palpable LNs during lateral neck dissection in patients with recurrent differentiated TC (63, 64).

Overall, SLNB demonstrates promise in improving the decision–making of siLNs, but the issues of a high false–negative rate and deficiency in tracer techniques persist (55). Nevertheless, continual upgrades are ongoing. For example, the innovative application of 68Ga-tilmanocept PET/CT has solved the longstanding “shine-through” problem in SLNB, which refers to the accumulation of peritumorally injected radiotracers in the tumor bed and has produced a large hotspot in imaging (65). Moreover, successful cervical SLN identification using 68Ga-tilmanocept PET/CT, especially in lateral compartments, has been demonstrated in a proof-of-concept study (66).

### 3.2 Intraoperative frozen section

Despite the various efficient techniques described above, there remain patients for whom the diagnosis of cervical small metastases using these methods proves hard. When a series of preoperative examinations of patients with small LNMs are clinically suspicious but not yet confirmed, the decisions of whether and to what extent to perform the dissection are complicated. In this scenario, after the possibility of local complications, surgical risks, and patient preference are balanced, intraoperative frozen section (FS) may be considered a last-resort diagnostic option. FS referred to a thorough regional LN dissection of the compartment suspected of metastasis based on anatomical or surgical boundaries, and then the specimens were sent to the pathology department for rapid testing (67, 68). All LNs within the compartment, including those smaller than 1 cm, are sent for frozen pathology examination, which helps alleviate the diagnostic challenges associated with the low volume of small LNs. Even if all the submitted LN specimens were less than 1 cm, the FS demonstrated excellent validity and consistency (69). Finally, according to the final FS report, the decision and scope of LND could be determined (the detailed instructions can be found in Section 4) (70).

At first glance, FS appeared to bear a striking resemblance to SLNB. There were, in fact, two noticeable differences between them: (1) the former requires whole-level dissection, whereas the latter solely involves the sampling of LNs stained with lymphatic channel dye, which are a portion of LNs within the entire compartment; (2) the purpose of the former is to determine whether extended cervical LND should be performed, whereas the latter is aimed at determining whether to remove the remaining LNs within the sampled level. According to current studies, FS seems to have a greater sensitivity and lower specificity in comparison to SLNB (67, 68, 71). A recent study indicated that FS could be replaced by preoperative FNAC combined with FNA-Tg because of its better diagnostic ability (67). Nevertheless, the observed outcome seemed to exhibit a degree of inconsistency with our actual experience derived from clinical practice. Collectively, FS pathology of the entire compartment was deemed to be a somewhat aggressive but pretty reliable diagnostic method. The term “aggressive” refers to underlying surgical complications such as nerve injury and lymphatic leakage (72), while “reliable” refers to the ability of FS to identify all LNs that require cleaning because patients who are negative in frozen sections or even positive in permanent sections are proven to be unnecessary for LND (73).

### 3.3 CK19-related techniques

CK19 is a type of cytokeratin with a relatively small molecular weight that is commonly expressed in both normal epithelial cells and epithelial-origin tumors (74). Obvious differences in expression can be observed between differentiated TC and benign thyroid lesions (75, 76).

At the mRNA level, a new biomolecular technique called one-step nucleic acid amplification (OSNA) can be used to quantify the number of CK19 mRNA copies (77, 78). Several studies have assessed the intraoperative diagnostic accuracy of OSNA for detecting LNMs in PTC patients (79–82). Although they incorporated LNs with varying diameters ranging from 3 mm to 17 mm, the vast majority of LNs were composed of LNs less than 10 mm in length, and OSNA showed a high level of accuracy for diagnosing indeterminate LNs. A meta-analysis incorporated several other studies and provided a pooled sensitivity of 88% and specificity of 90% (83). This result was derived from a comprehensive analysis of all LNs, regardless of size. Additionally, a positive linear relationship between CK19 expression and LN size was proven in a prospective study (81).

At the protein level, cytokeratin fragment 21-1 (CYFRA 21-1) is a proteolytic portion of CK-19 and has been proposed to be a dependable biomarker owing to its high expression in the extracellular fluid (84, 85). Lee et al. (86) reported that washout CYFRA 21-1 measurements in indeterminate LNs served as a valuable diagnostic approach, by which 91% of LNs with discordant FNAC and FNA-Tg results were accurately confirmed. Moreover, a new technique termed immunochromatographic strip significantly decreased the detection time of CYFRA 21-1, which has dramatically increased the application prospects of the approach (87). Despite its great potential, the effectiveness of CYFRA 21-1 measurements still lacks additional validation in patients with siLNs.

## 4 Potential siLNs detection techniques used in other types of tumors

**Figure 3:**
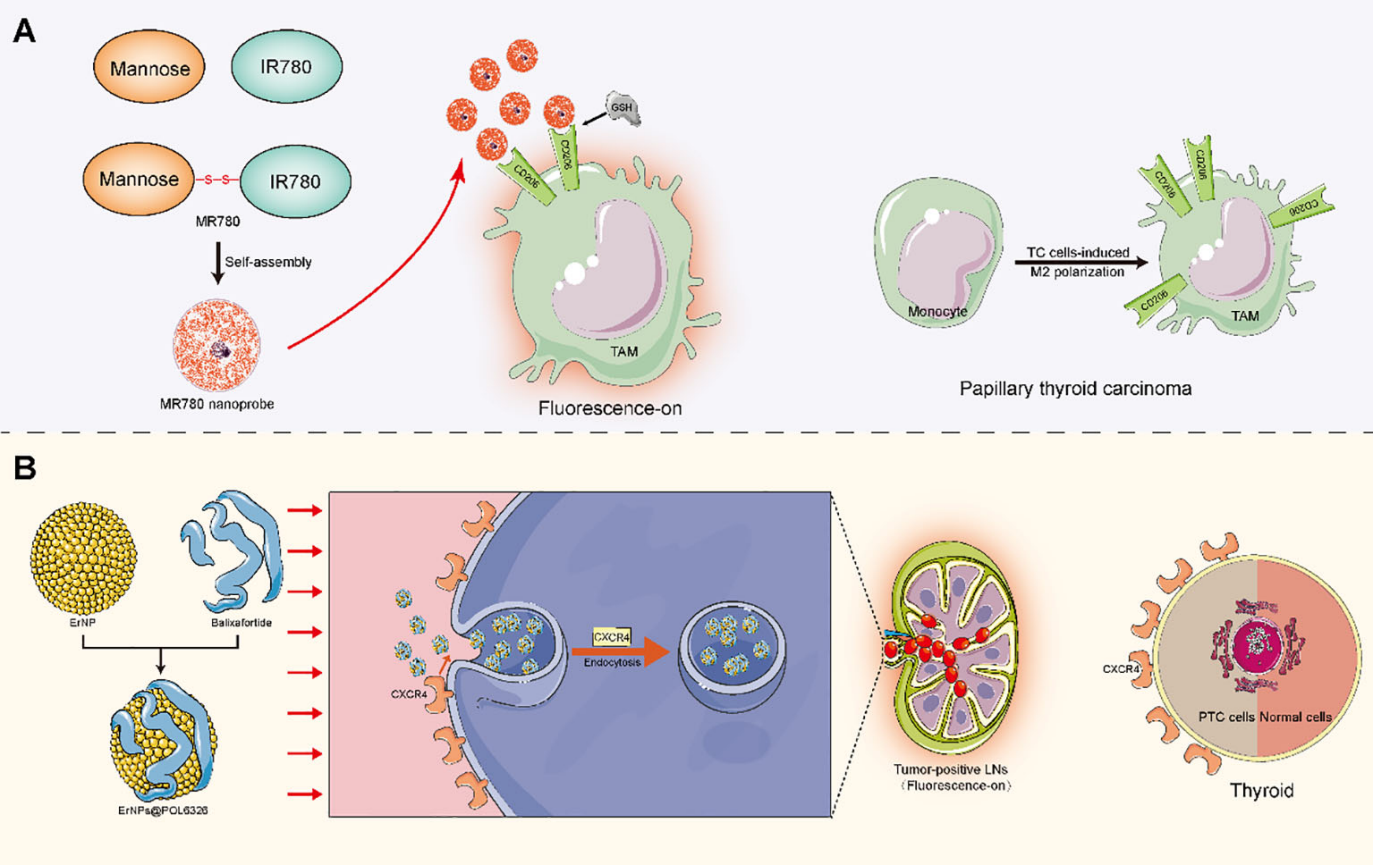
Mechanistic diagram of potential detection techniques for siLNs. **(A)** Mannose was disulfide bonded with the near-infrared dye IR780 to form a Mannose-IR780 conjugate (MR780), which was further self-assembled into quenched fluorescence nanoprobes. The ligand Mannose exhibited specific affinity for the CD206 molecule, which is a highly expressed receptor on the TAM surface. When CD206 is selectively targeted, the disulfide bond is cleaved under the influence of the microenvironment (including GSH), thus achieving fluorescence molecular imaging of micrometastasis through interactions between specific dyes and molecules. **(B)** The ErNPs were modified by balixafortidea, a peptide antagonist of the chemokine receptor CXCR4, to assemble the fluorescence probe ErNPs@POL6326. The probes could be efficiently drained into LNs when given subcutaneously to infiltrating metastatic breast tumor cells via CXCR4-related endocytosis. A significant difference in fluorescence signals was observed between metastatic LNs and nonmetastatic LNs. TAM, M2 tumor-associated macrophage; GSH, glutathione; TC, thyroid cancer; PTC, papillary thyroid cancer.

Aside from the methods mentioned above, other viable alternative methods should be identified. Diagnostic techniques that work well for other types of tumors may also potentially yield favorable outcomes in PTC patients. For instance, the aforementioned contrast agent Sonazoid, which was previously used in the detection of liver lesions, has demonstrated exceptional performance in identifying PTC-related siLNs (20, 21, 88). In breast cancer, Zhao et al. (89) developed a targeted fluorescence imaging method based on M2 tumor-associated macrophages (TAMs) for noninvasive and precise detection of micrometastatic LNs (shown in **Figure 3**). Notably, TC cells can induce human monocytes to undergo M2-like polarization, leading to a significant increase in CD206 expression (90). Similarly, Zhu et al. (91) constructed multicomponent rare-earth nanoparticle probes to infiltrate and label metastatic breast tumor cells through CXCR4-related mechanisms for NIR-IIb fluorescence bioimaging (shown in **Figure 3**) (92). Indeed, benign thyroid lesions have low or no CXCR4 expression, whereas most PTCs do (93). In this way, this PTC-related evidence seems to suggest that the aforementioned techniques may be suitable for detecting siLNs in PTC patients. Currently, the advent of nanomedicine technology has also led to the development of innovative approaches for accurate imaging of tiny LNs (94). As such, a clinical trial showed that the utilization of superparamagnetic iron oxide nanoparticles in MRI significantly boosted the ability to detect LNMs measuring less than 5 mm in cases of rectal or prostate cancer (95). The use of nanoparticle-enhanced MRI (even of the same magnetic nanoparticles) for visualizing PTC-related LNs has been studied, but whether this approach works well in the diagnosis of siLNs is unclear (96). Accordingly, on the premise of ensuring the feasibility, other alternative methods with excellent diagnostic power should be considered for PTC patients.

## 5 The management of siLNs: superselective dissection and active surveillance

**Figure 4:**
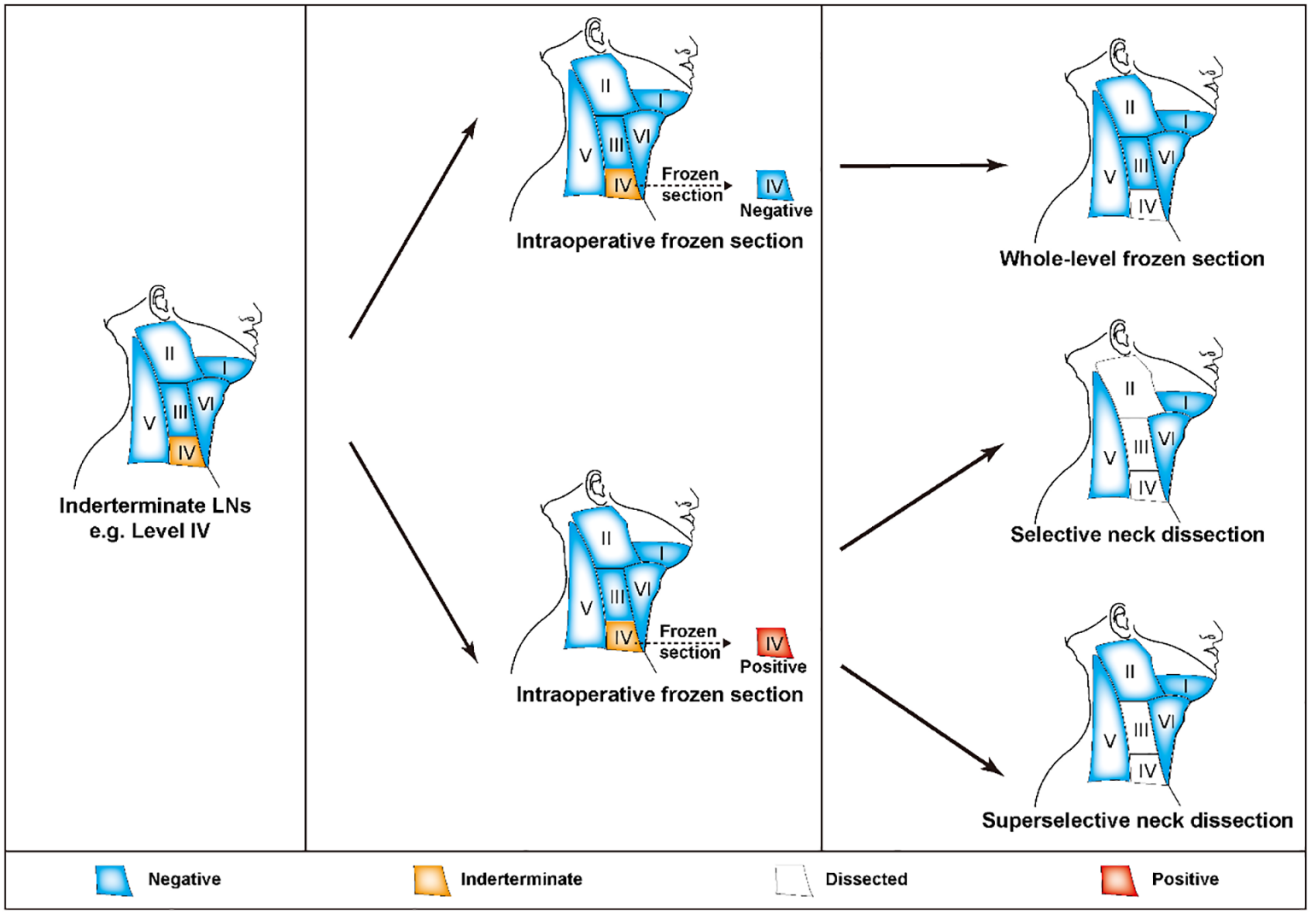
FS-assisted surgical decision-making in the management of lateral cervical siLNs. If there were any siLNs in compartment IV, the patients were first subjected to single compartment dissection at level IV. The following removal scope was determined based on the results of FS analysis. If there was no metastasis, the current degree of lymphadenectomy was sufficient. If metastasis was proven, levels II-III or III dissection was suggested according to whether the patient underwent SND or SSND, respectively. FS, frozen section; siLNs, small and indeterminate lymph node; SND, selective neck dissection; SSND, superselective dissection.

In general, lateral cervical metastasis most commonly occurs in compartments II-IV, as do siLNs (97). According to the current guidelines established by the ATA, “patients with biopsy-confirmed metastatic lateral cervical lymphadenopathy should undergo therapeutic lateral compartmental LND” (10). However, some scholars argue that LND that includes only the level of FNA-proven metastasis is insufficient (98, 99). The current controversy revolves around the exact extent of lateral neck dissection, and a recent review has provided a detailed description of this topic (100). Indeed, preoperatively metastasis-confirmed siLNs can be considered typical cervical LNMs, and the dissection scope should be selected according to the established routine criteria described in the aforementioned review (100). For these undiagnosed siLNs, superselective neck dissection (SSND) in conjunction with FS is a useful method that can achieve more precise diagnosis and treatment. The term referred to the surgical removal of LNs, adipose, and fibrous tissue in one or two adjacent neck compartments. Initially, employed for the treatment of early-stage head and neck cancers, SSND is presently used in the management of lateral cervical siLNs of PTC (101, 102). Take level IV as an example, when siLNs were found in this compartment, level IV dissection was performed first. The decision to perform successive LNDs was based on the FS analysis at level IV. If the result was negative, the extent of lymphadenectomy was sufficient, and the remaining compartments were preserved. In contrast, if the lesion was positive, subsequent dissection of levels II and III was recommended according to the perspective of SND, whereas removal of level III alone was deemed sufficient according to the perspective of SSND (**Figure 4**) (10, 101, 103, 104). Actually, to perform a level II dissection did not influence the following treatment decision, nor did it result in a statistically significant difference in recurrence rates (105). Therefore, SLNB should be regarded as a potentially promising alternative due to its advantages, which include the use of smaller incisions and fewer complications. However, if some high-risk factors for recurrence, such as excessive LN yield and extranodal extension, are present, a more radical surgical method that involves LND of compartments II-IV may be considered more suitable (106, 107). In fact, FS assessment of nodal status was also taken into consideration for the decision of extended central LND (108). When the presence of ipsilateral central neck and pretracheal-laryngeal occult LNM was established through FS analysis, prediction models could assist in identifying patients who would benefit from contralateral dissection (109, 110).

Previous publications have suggested that cervical siLNs detected after initial surgery can be followed up under AS (11, 111). It was reported that a mere 16.9% of patients necessitated invasive intervention, suggesting the potential for emulating this approach to finish the management of siLNs before initial surgery (112). Nevertheless, studies on whether preoperative siLNs can be treated with AS in PTC patients or only in those who have been properly selected are lacking (30). siLNs was deemed not advisable to proceed with FNA because of the low success rate associated with the small volume of the lesions (40, 41). If successful, a patient with a negative result can undoubtedly proceed with AS based on ATA2015 guideline (10). In this scenario, AS is a more conservative strategy than SND or SSND. The advantages of AS mainly include the following: (1) no additional surgical risks or postoperative problems associated with LND and (2) without considering the impact of LNM-related factors on prognosis, revision dissection could also achieve a good surgical outcome due to the absence of postoperative adhesions. Last, the patients treated with AS require more frequent medical review than those undergoing usual postoperative management.

## 6 Summary and outlook

Typically, PTC is regarded as an indolent form of tumor with a favorable prognosis. However, a considerable portion of patients experience adverse clinical outcomes, including LNM and lymphatic recurrence. Therefore, close attention should be given to the detection and management of cervical LNs in the process of diagnosis, treatment and follow-up. siLNs are commonly recognized during preoperative evaluations, but determining their nature is a significant challenge owing to their limited size and borderline characteristics. With the rapid development of medical imaging and molecular medicine, the diagnostic difficulties associated with siLNs have gradually improved due to the utilization of a series of novel techniques and methods, including CEUS, CT parameter modeling, FNA-Tg, FS, and molecular detection. These tools have the capacity to provide guidance for clinical management, in terms of selecting appropriate surgical interventions or AS, as well as determining the optimal extent of surgery, ultimately facilitating patient-beneficial decision-making and improving PTC prognosis.

Preoperatively, techniques associated with US, CT, and FNA have been recognized by published studies as effective ways to evaluate cervical siLNs. In this field, preliminary CEUS research has shown encouraging outcomes, but corresponding clinical translation is still a major challenge because the sample sizes of these studies were too small to reach a definitive conclusion. Moreover, the injection site and the contrast agent may need further improvement to solve the problem that the imaging waiting time of compartments III and V was too long (20, 21). Compared with the utilization of US or CT alone, their combination with emerging radiomics has markedly enhanced the diagnostic and predictive efficiency of high-throughput extraction of large numbers of imaging features (113, 114). To the best of our knowledge, studies have yet to develop a radiomics-based model with US and/or CT images to specifically assess siLNs in patients with PTC. Therefore, radiomics may serve as an important development direction for future research in this respect, as does the imaging parameter model. Most of the currently widely-accepted guidelines do not offer a detailed illustration of the indications for FNA biopsy of LNs, including the LN size and US and CT signs. Even if such standards were mentioned, they were also significantly different (**Table 1**). In addition, there is no well-recognized definition of “small” for cervical LNs in PTC patients. Both 8 mm and 10 mm have been adopted for previous studies (21, 26). Thus, deciding on a clear definition of “siLNs”, investigating when to conduct the FNA, and ultimately concluding a uniform consensus are necessary.

Intraoperatively, the available options, including SLNB, FS, and CK-19 testing, may be the last resorts for diagnosing siLNs. SLNB often prevents patients from undergoing more invasive surgical procedures by confirming the absence of metastasis in the sentinel nodes. Therefore, a practical SLN definition that takes the lymphatic drainage junction into account is urgently needed to assist surgeons in accurately determining which LNs should be fully removed to ensure that real biological SLNs are sent for pathological examination. However, SLNB is currently not a routine procedure and its effectiveness needs to be further verified within the scope of PTC. Like the combination of BRAF and FNA for preoperative diagnosis, CK-19 combined with FNA, SLNB, or FS (FNA+CK-19, SLNB+CK-19, or FS+CK-19) is also of potential value for the auxiliary diagnosis of siLNs during surgery. In China, despite patients being informed of the applicability of follow-up and revision surgery, the majority of patients prefer to solve the problem once and for all, which is probably influenced by traditional beliefs about malignancy. Thus, FS, at least that appear fairly radical and reassured from a patient perspective, can fulfill their requirements to a great extent by dissecting and examining all suspicious compartments with siLNs. Meanwhile, FS can reliably identify patients who would benefit from a more extensive surgical procedure, thus decreasing the likelihood of recurrence or a second operation. Compared to SND, SSND reintroduces a degree of conservatism to aggressive FS analysis by virtue of a much shorter incision, a smaller dissection extent, and less surgical risk. In future studies, we will need further information concerning the range of applications where selective LNDs can be replaced with superselective LNDs. Facing challenges such as economic cost, follow-up protocols, compliance, and patient anxiety, research on AS of siLNs is still in its infancy, in which one thing that cannot be ignored is to increase public knowledge about the indolence of PTC.

**Figure 5:**
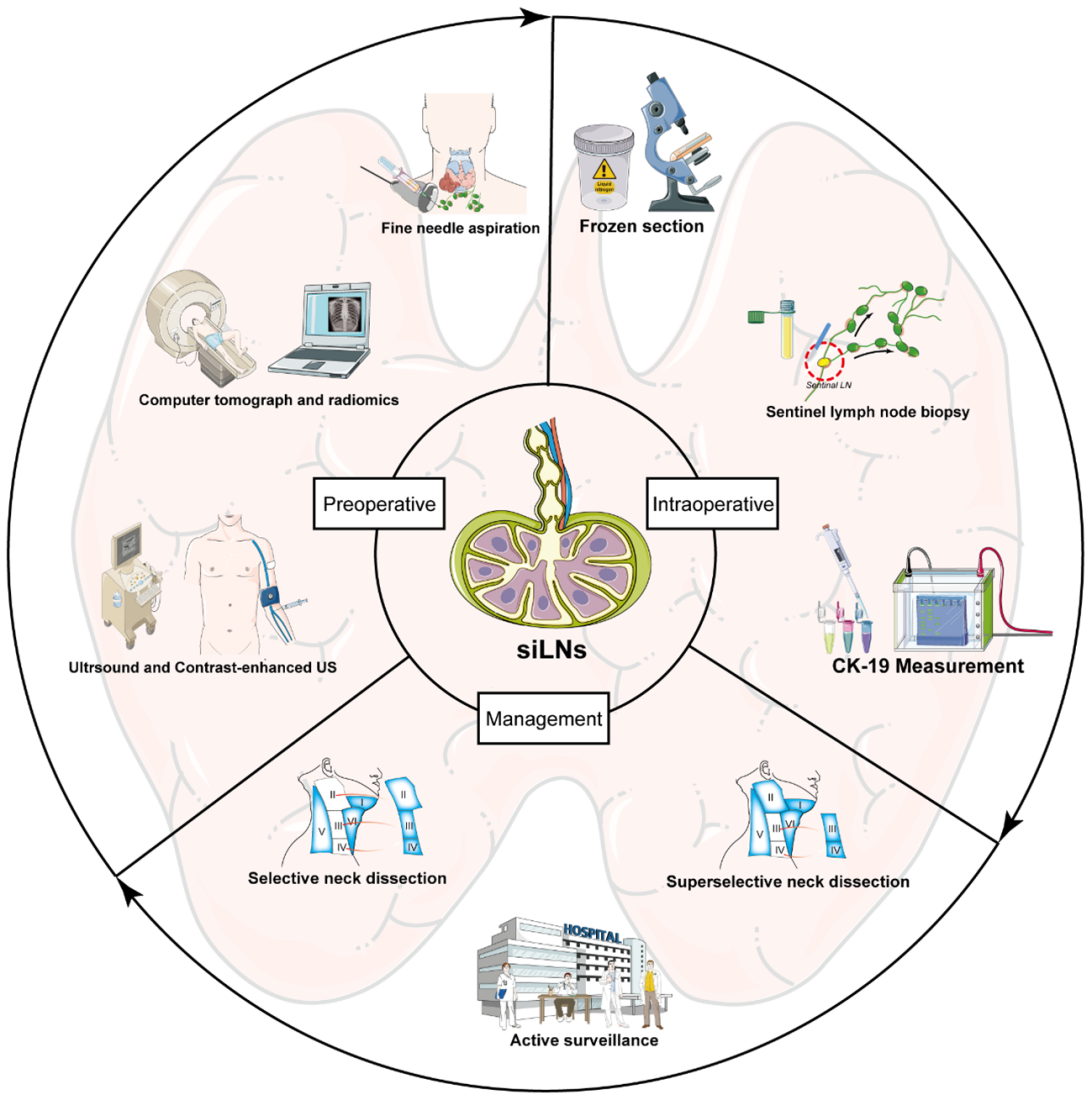
A schematic diagram throughout the entire process of diagnosing and managing siLNs.

In conclusion, the presence of siLNs poses a great challenge in clinical practice and substantially exacerbates the medical burden of PTC. According to existing studies and expert opinions, we offered a comprehensive and updated overview of feasible approaches for the diagnosis and management of siLNs (summarized in **Figure 5**). To the best of our knowledge, this is the first systematic review focusing on small cervical LNs in the field of PTC. We believe that by combining conventional methods and cutting-edge technology, precise assessment of siLNs in PTC patients is possible, as is the provision of more standardized guidance that could optimize clinical decision-making, ultimately resulting in improved patient outcomes and quality of life.
